# Mapping evidence of nurses’ attitudes toward older adults in Africa: a scoping review protocol

**DOI:** 10.1186/s13643-021-01575-y

**Published:** 2021-01-09

**Authors:** Esther L. Wanko Keutchafo, Jane Kerr, Mary A. Jarvis, Desmond Kuupiel

**Affiliations:** 1 Discipline of Nursing, School of Nursing and Public Health, University of KwaZulu-Natal, 71 Manor Drive, Manor Gardens, Durban, 4001 South Africa; 2 Research for Sustainable Development, Sunyani, Ghana

**Keywords:** Africa, Attitudes towards older adults, Nurses, Nursing students

## Abstract

**Background:**

Culture influences nurses’ attitudes towards caring for older adults. Additionally, nursing students’ perceptions and attitudes towards older adults affect their behavior, possibly their career choices and/or the quality of care provided to older adults after graduation. In the context of lower–middle-income countries with a faster growing older adults population compared to upper income countries, the improvement of the quality care, inclusive of nurses’ attitudes towards older adults, is one of the strategies for strengthening nursing and midwifery in Africa. Furthermore, examining nurses and nursing students’ attitudes towards older adults will answer the United Nations’ call for more data to understand the needs and the status of older adults in Africa.

**Methods:**

This scoping review will be guided by Arksey and O’Malley’s framework. The search will be performed using Scopus, PubMed databases, Academic search complete, CINAHL with full text, Education source, Health source: Nursing/Academic Edition, with words related to the topic. The reviewers will also use Google Scholar and the reference lists of the relevant articles. Primary studies and grey literature addressing the research question will be included. The search process will include a first stage where two reviewers will perform the title screening and the removal of duplicates, followed by a parallel abstract screening according to eligibility criteria. The second stage will involve the reading of full articles and the exclusion of articles, in accordance with the eligibility criteria. Data will be collated by two reviewers independently and parallel, using a predetermined data extraction form. Discrepancies will involve a third reviewer. The Mixed Methods Appraisal Tool, version 2018 will be used to assess the quality of the data of eligible articles. A narrative approach containing summary tables and graphs will facilitate synthesis.

**Discussion:**

The review will provide insight into nurses' and nursing students’ attitudes towards older adults in African countries. The outcomes will guide future research, practice, and education in nursing.

**Supplementary Information:**

The online version contains supplementary material available at 10.1186/s13643-021-01575-y.

## Background

Globally, every country is witnessing a growth in the number and proportion of older adults [1–5]. In 2019, evidence showed that older adults, aged 65 years or over, were 703 million and numbers are expected to reach 1.5 billion in 2050 worldwide [5]. In 2010 in Africa, it was estimated that older adults represented 3.6% of the population [6], with figures increased in 2019 to 32 million in sub-Saharan Africa, and projected to reach 101 million in 2050, an increase of 218% [5]. Although ageism is not a universal stigmatizing behavior, it has been reported throughout civilization, in particular among younger people [7], and have elicited stereotypes about both the reality and the circumstances of old age [8]. In Africa, relations between younger and older adults were marked by respect for the latter by the former; however, more recently, younger people are suggested to ignore or exclude older adults [2], framed within ageism [9].

Concern needs to be directed towards older adults in Africa because sub-Saharan Africa has one of the most rapidly growing older populations when compared to any other region in the world [5, 10]. Older adults play a role in securing livelihoods and contribute towards social and economic development outcomes [11]. Moreover, the older population of yesteryear will not be the same as the one of the future [12], as they will use healthcare services more frequently than the younger population [13] because their healthcare needs tend to be more complex and chronic [14]. Therefore, healthcare reforms have to take into account the resourcing of services to meet the healthcare needs of older adults [15]. The direct implication is that more healthcare workers will be required to spend more of their working time with older adults [16]. Yet, older adults require specialized nursing knowledge, skills, and attitudes, to achieve positive health outcomes and prevent a variety of geriatric syndromes [17–19]. Consequently, nurses, as the largest proportion of healthcare workers [20], should not see older adults in stereotyped ways with ageist and paternalistic attitudes [21], but should have positive attitudes towards them.

Attitudes can be viewed as the expression of beliefs, feelings, and experiences with regard to an object or concept, which are reflected through cognition and affection, and influence behaviors [22]. In relation to students, attitudes towards older adults are patterns of feeling and beliefs held either in positive or negative ways [23]. Nursing students’ attitudes towards older adults and aging are likely to affect their nursing behaviors [24] and both their career choices and the quality of care provided to older adults [25–29]. Yet, gerontological nursing education can play a significant role in fostering the development of positive attitudes towards older adults [21, 30]. Therefore, the possible identification of negative attitudes towards older adults should be a concern for nurse educators [31].

Reviews have been conducted incorporating nurses' and nursing students’ attitudes towards older adults. In 2013, Liu et al. [32] conducted a systematic review on registered nurses’ and nursing students’ attitudes towards older adults and the potential underpinning variables reflected in studies from 2000 to 2013. Three studies conducted in African countries were reported. An Egyptian study used four different scales including an Arabic version of the Kogan’s attitude towards older people (KOP) scale [33], while a Malawian study involving medical and nursing students also used the KOP scale [34]. A Nigerian study used a 30-item questionnaire developed by the researchers after an extensive literature review [35]. In 2014, Neville and Dickie [36] conducted a literature review including publications between 2008 and 2013, which aimed at evaluating undergraduate nurses’ attitudes toward older adults and their perceptions of working with older adults. Only one study conducted in Malawi was reported [34]. In 2017, Hovey et al. [30] conducted an integrative review synthesizing empirical studies from 2009 to 2015 from the USA and Canada to gain an understanding of how nursing education affects nursing students’ attitudes toward older adults. Although the two reviews which included African studies were done on attitudes towards older adults, they were not exclusive to African countries. The first was non-geographically specific while the second review was conducted in the USA and Canada only.

Over the years, despite a global interest in attitudes, perspectives, and perceptions of nurses’ attitudes towards older adults [37], there is no study carried out to systematically review studies focused on nurses’ and nursing students’ attitudes towards older adults in African countries exclusively. Yet, there is a call for more data to understand the needs and the status of older adults in Africa [38] because most of the evidence-based data related to geriatric health needs are from populations in high-income countries [10]. To answer the call, this scoping review is nested in a larger study aiming at analyzing nonverbal communication between nurses and older patients to develop a model of effective nonverbal communication between nurses and older patients. Therefore, this review intends to systematically review evidence examining nurses' and nursing students’ attitudes towards older adults in African countries to contribute towards bridging the gap.

## Methods

A scoping review is planned instead of a systematic review because the aim of the study is to map evidence on nurses' and nursing students' attitudes towards older adults in Africa. To be specific, this review is to examine how research is conducted on the topic, rather than to confirm or refute stereotypes of attitudes towards older adults, or to address any uncertainty or variation on how to assess attitudes towards older adults that may be occurring [39]. The study will adopt the framework proposed by Arskey and O’Malley [40] and further refined by Levac et al. [41]. In summary, the framework involves the following: identification of the research question and of the relevant studies, study selection, chart of the data, collation, summary, and report of the results. Additionally, the Preferred Reporting Items for Systematic reviews and Meta-Analyses extension for Scoping Reviews (PRISMA-ScR) guidelines [36] was used to prepare this protocol (Additional file 1).

### Identification of the research question

The research question will be what are the documented nurses' and nursing students’ attitudes toward older adults in Africa? The Joanna Briggs Institute PCC (population, concept, context) mnemonic was used to determine the eligibility of the scoping review question as suggested by Peters [42]. It is illustrated below (Table 1).

**Table 1:** The population, concept, context mnemonic to identify the research question

Category	Inclusion criteria	Exclusion criteria
Population	Nurses, including nursing students	Other healthcare workers Other healthcare students Other healthcare professionals Informal caregivers
Concept	Attitudes towards older adults	Knowledge about older adults Perceptions of older adults Care of older adults
Context	African countries	Non-African countries

Studies meeting the following elements will be included in the study:

- Studies aiming at nurses’ attitudes toward older adults in African countries;
- Studies aiming at nursing students’ attitudes toward older adults in African countries;

Studies with the following elements will be excluded from this study:

- Studies aiming at nurses’ knowledge, perceptions, and care of older adults in African countries;
- Studies aiming at nursing students’ knowledge, perceptions, and care of older adults in African countries;
- Studies with the above inclusion criteria but focusing on non-African countries.

### Identification of the relevant studies

An extensive range of sources will be used to ensure comprehensive coverage of the literature. The electronic databases to search for relevant articles will include academic search complete, CINAHL with full text, Education Source, Health Source: Nursing/Academic Edition, via EBSCOhost search engine. Studies will also be searched from Web of Science, PubMed, and Google Scholar databases as well as the reference lists of the included articles to identify more  relevant literature.

The search terms for this review originated from indexed subject headings, keywords of relevant studies, terms from this review protocol that recurred repetitively and the Medical Subject Headings (MeSH) terms. The string/Boolean search terms for this review will include (nurse(s) OR nursing student(s) OR student nurses OR undergraduate nurse) AND (attitudes OR perceptions OR opinions OR thoughts OR feelings OR beliefs) AND (old people OR elder OR elderly OR older people OR aged OR geriatrics OR seniors OR aged 65 or 65+). Additionally, the symbol * will be placed after the root of the search terms to broaden the search to include various word endings and spellings. Any study design will be included in the study. Quantitative studies will include randomized controlled trials, non-randomized controlled trials, quasi-experimental studies, before and after studies, analytical, and descriptive cross-sectional studies. Qualitative studies will include case studies, grounded theory, phenomenological, and ethnographic studies. Mixed methods as well as reviews and grey literature will also be included. Language limitations will be removed during the search. An initial search was performed in CINAHL with full text. The results are displayed in Table 2.

**Table 2:** Initial search

Keyword search	Date of search	Engine used	Number of articles retrieved
(attitudes or perceptions or opinions or thoughts or feelings or beliefs) AND (nursing students or student nurses or undergraduate student nurses) AND (older adults or elderly or geriatric or geriatrics or aging or senior or seniors or older people or aged 65 or 65+) Published date: 20000101-20191009 Source types: academic journals	09 October 2019	CINAHL with full text through Ebscohost	1059

### Study selection

The selection of studies will involve two stages. The first stage will involve title selection from the proposed databases by EW and JK independently to determine the eligibility of the study based on the inclusion and exclusion criteria. The identified articles will be imported to Endnote X9 reference management software. Next, duplicates will be removed before EW and JK independently screen the abstracts and the full text of the eligible articles in parallel. The University librarian will be consulted to assist in locating and providing articles without full texts. Emails will be sent to authors to request for identified relevant articles where full texts are not retrievable. Should discrepancies arise between EW and JK, they will be first resolved through discussion until a consensus is reached. If no agreement is obtained, the third reviewer, MAJ will be involved and her decision will be final at both abstract screening and full-text screening stages. A French reviewer will be sought to co-screen with EW, the abstracts and the full-texts of the eligible articles published in French. A collaboration will be built, with the study University Systematic Review Unit to screen other studies that may be published in other languages apart from English and French. Additionally, although a library scientist was not involved in developing the search strategy, one will be consulted if there is a need. All the screening will be performed using Google forms. Reasons for excluding articles will be recorded and reported. A PRISMA flow diagram summarizing the search and screening processes will be presented in the final review.

### Data extraction

A draft data extraction form has been developed at this stage to aid the collection and the sorting of key information from the included studies (Table 3). Data to be extracted will include information such as author(s), year of publication, study objective, country, study design, study population, study findings, and significant outcome(s). Additionally, specific information about study samples, such as age of participants, religious and/or cultural background, and geographic context, as well as tools used to assess attitudes towards older adults will be extracted. The data extraction form will be piloted prior to its final usage to address discrepancies on a random sample of four included articles. The form will be updated continuously during this phase of the review to capture all required and relevant data based on feedback from EW and JK. The authors will be contacted for clarification, if there is any missing or inadequately reported data.

**Table 3:** Initial data extraction form

**Bibliographic information**	
Author(s) and year	
Article title	
Country	
**Methodology**	
Study aims	
Study design (quantitative, qualitative, mixed-methods)	
Population (nurses or nursing students)	
Sample size	
Setting (nursing schools, hospitals, communities, other)	
Geographic location (urban, rural, both)	
**Tool(s) used (when applicable)**	
Validity	
Reliability	
**Total attitudes (**positive, negative, or neutral**)**	
Attitudes score	
**Themes described**	
**Other relevant information**	
**Conclusion**	

### Quality assessment

All the included primary studies will be subjected to rigorous appraisal to assess the methodological quality of a study and to determine the extent to which a study addressed the possibility of bias in its design, conduct, and analysis [43] though it is not a requirement of scoping reviews [44]. For the quality appraisal, the Mixed Methods Appraisal Tool (MMAT), version 2018 [45] will be independently used by EW and JK as it is a critical appraisal tool designed to assess quality of reviews that include qualitative, quantitative, and mixed-methods studies [45]. Discussion will be used to resolve discrepancies. The MMAT will allow assessment of the appropriateness of the aim of the study, methodology, study design, participant recruitment, data collection, data analysis, and the findings presented. The quality of studies will be graded with a quality score ranging from ≤ 50% as low quality, 51–75% considered as an average quality, to 76–100% considered as high quality.

### Collation, summary, and reporting the results

Content analysis will be employed to abstract relevant data, its meaning, and text will be summarized, manually coded into overall categories [46] independently by EW and JK. NVivo version 12 will be used to organize and manage data from the included studies. A narrative account will present the findings from existing literature discussing attitudes towards older adults, the different tools used to assess the attitudes as well as the characteristics significantly associated with the attitudes. It will be presented in a descriptive format that will seek to investigate similarities and differences between studies to explore patterns, themes, and relationships and propose explanations for findings. The results of this scoping review will be presented following the Preferred Reporting Items for Systematic Reviews and Meta-Analyses extension for Scoping Reviews (PRISMA-ScR) guidelines [47].

## Discussion

This study will respond to the United Nations call for more data to understand older adults in Africa [38]. Reviewing African nurses' and nursing students’ attitudes towards older adults will speak to one of the global strategic directions for strengthening nursing and midwifery in Africa [14]. The results will also provide evidence-based knowledge, inform future research, and enrich the main study’s findings. Characteristics associated with negative or positive attitudes towards older adults will be identified.

Owing to the fact that culture influences attitudes towards caring for older adults [24], there is a need to conduct a study on nurses' and nursing students’ attitudes towards older adults in African countries. In African cultures, respect for older adults remains a notable tradition; however, due to socioeconomic and cultural changes caused by modernization and urbanization, it seems to be likely that older adults are either isolated or excluded within African societies [2]. Therefore, it is expected to find both positive and negative attitudes displayed towards older adults. It is anticipated that a follow-up study (meta-analysis) using the quantitative data obtained from this proposed scoping review will be conducted to assemble similar quantitative studies into a single estimate. Attitudes towards older adults affect career choice, behavior, and geriatric care [24, 26, 29]. Therefore, identified characteristics associated with negative attitudes will inform the modification of curricula to develop gerontological nursing in order to decrease nurses' and nursing students’ misconceptions of older adults and improve their attitudes [48] or to design gerontological content and clinical experiences to foster positive attitudes towards older adults [30].

However, the reviewers anticipate some limitations, although rigorous steps will be followed throughout this review. Firstly, studies not published in the selected databases may be omitted from the review. Secondly, other healthcare workers’ attitudes towards older adults will not be described.

## Supplementary Information

**Additional file 1:.** Preferred Reporting Items for Systematic reviews and Meta-Analyses extension for Scoping Reviews (PRISMA-ScR) guidelines

## Data Availability

All data generated or analyzed during this study in the published scoping review will be included.

## Abbreviations

MMAT: Mixed methods appraisal tool
PCC: Population, concept, context
PRISMA: Preferred reporting items of systematic reviews and meta-analyses
PRISMA-ScR: Preferred reporting items for systematic reviews and meta-analyses extension for scoping reviews
WHO: World health Organization
