# Supplementary material for: Mapping evidence of nurses’ attitudes toward older adults in Africa: a scoping review protocol
Source: Syst Rev. 2021 Jan 9;10:19. doi: 10.1186/s13643-021-01575-y (PMC7797139; doi:10.1186/s13643-021-01575-y)
Supplement: Supplementary file 1 — Additional file 1:. Preferred Reporting Items for Systematic reviews and Meta-Analyses extension for Scoping Reviews (PRISMA-ScR) guidelines [file 13643_2021_1575_MOESM1_ESM.docx]

**Point-by-point response to reviewers**

Thank you very much for the review of our submission and for your valuable feedback. The table below addresses your suggestions.

| **Reviewers comments** | **Effected changes** | **Line numbers** |
| --- | --- | --- |
| **Reviewer 1** | | |
| There are still some inconsistencies in the description of the aim and scope of the review:  Line 101: "(…) this review intends to systematically review evidence examining nurses and nursing students' attitudes towards older adults in African countries." Focus on an analytic approach, a systematic review will be appropriate. | Thank you for bringing to our attention the confusion. The authors have realized that the most appropriate methodology would be a scoping review in order to map the evidence. | 109 - 110 |
| Line 105: "(…) the aim of the study is to map evidence on nurses and nursing students attitudes towards older adults in Africa to examine how research is conducted on the topic, and to identify characteristics associated to attitudes towards older adults rather than to confirm or refute stereotypes of attitudes towards older adults, or to address any uncertainty or variation on how to assess attitudes towards older adults that may be occurring". "Mapping the evidence" and "identify characteristics associated to attitudes towards" are different aims, the first one is for a scoping review, the second one for a systematic review | The authors changed the aim of the study to “*the aim of the study is to map evidence on nurses' and nursing students' attitudes towards older adults in Africa to examine how research is conducted on the topic, rather than to confirm or refute stereotypes of attitudes towards older adults, or to address any uncertainty or variation on how to assess attitudes towards older adults that may be occurring*.” | 109 - 114 |
| Line 117: "The research question will be: What are the documented nurses and nursing students' attitudes toward older adults in Africa?" The analysis of the associated characteristics is not mentioned here.  Please clarify the aim, scope and objective of the review throughout the protocol and choose the appropriate review method. | The authors do not intend to statistically analyze the associated characteristics of attitudes toward older adults as it pertains to systematic reviews and not to scoping reviews. Therefore, all the mentions related to associated characteristics were removed throughout the manuscript. | 111 - 112 183 - 184 |
| Title: please use either "scoping review" or "systematic review" based on your final decision, rather than "systematic scoping review". | After the final decision, the authors used the term scoping review in the title. | 2 |
| Line 126 exclusion criteria: "Studies aiming at nurses' knowledge, perceptions (…) of older adults". It seems quite difficult to distinguish between attitudes and perceptions. "Perceptions" is also mentioned as a search term in the protocol (table 2).  Please add some more details about the concept of attitudes in this review. Please clarify also whether instruments assessing perceptions and opinions as one aspect of attitudes will be included or excluded. | Thank you for your comment. As described in the background, attitudes are expressions of beliefs, feelings, and past experiences regarding an object/concept and are reflected through cognition and affection influencing behaviors (Lee, 2009). When perceptions are associated with positive/negative connotations, they become attitudes (Sarabia-Cobo & Pfeiffer, 2015). These show that attitudes and perceptions are somehow linked. However, the study will only include studies on attitudes towards older adults. If a study reports on both attitudes and perceptions, only results on attitudes will be included. |  |
| Table 3: please check and add relevant information about the data extraction for qualitative studies. | Data extraction for qualitative studies will include findings reported in the studies. The mention “themes described” was added in the data extraction form. | Table 3 |
| **Reviewer 2** | | |
| In the Abstract the JBI method is still mentioned. | The JBI method was deleted from the abstract. | 28 - 29 |
| Line 157: The de-duplication is still planned after the title screening but is not clear why title lists including duplicates will be screened in the first step. | The titles’ list including duplicates will not be screened in the first step. The two authors will select the titles instead. This mistake was corrected under study selection. | 158 - 159 |
| **Reviewer 3** | | |
| On page 4, the authors have listed additional studies of systematic reviews that are relevant to their topic, but they do not state the results, strengths, limitations, or how the research provides the case for an additional scoping review. | The authors listed systematic reviews conducted on the topic, as suggested by one of the reviewers. Though the two reviews were done on attitudes towards older adults, they were not exclusive to African countries. The first was non-geographically specific while the second review was conducted in the USA and Canada only. The United Nations calls for more data to understand the needs and the status of older adults in Africa, hence this proposed study is envisaged to contribute towards bridging the gap. | 93 – 95; 107 |
| 2. On the same page, the authors discuss the KOP scale--please spell this out the first time an abbreviation is used. Further, I'm not sure how the scale is relevant to their scoping review. | The KOP stands for the ‘Kogan’s attitude towards older people’ scale and was spelled out the first time.  Though the KOP scale was mentioned, it is part of the numerous scales that have been used to examine attitudes towards older adults. The other scales are the Aging Semantic Differential, The Facts on Aging Quiz, the Aged inventory, the Perceptions of Working with Older People scale, and the McLafferty scale (Liu, Norman, & While, 2013). Yet, the KOP was the first scale to be developed (Urbanová & Bužgová, 2017), and has been used to explore professional and student healthcare workers ATOPs (Flores, 2016).  In this review, any scale used to examine attitudes towards older adults will be extracted as mentioned under data extraction. | 84 |
| 3. On page 5, the authors state that there is no meta-analysis that addresses their topic, but the purpose of the research that they will report is for a literature review, not a meta-synthesis. | The mention meta-analysis was deleted in the background as this current report is a scoping review. | 97 - 98 |
| 4. On page 5, the authors state that the scoping review is nested in a larger study, but there is no description of that study. Please add the details as to how the review they present is part of the study, and details of the study itself. | The larger study aims to identify nurses’ nonverbal communication strategies used when communicating with hospitalized older adults to develop a model of effective nonverbal communication between nurses and older patients. The initial results showed some positive attitudes towards older adults in Cameroon (Wanko Keutchafo and Kerr, 2020a). One recommendation for future research was to examine attitudes towards older adults in African countries to strengthen the model development centred on nonverbal communication which is part of patient-centred care. Though these details are provided as requested by the reviewer, the authors did not include the initial results in this report because the study itself is not yet complete. |  |
